# Multiple screws versus sliding hip screws in femoral neck fractures: A protocol of cohort study

**DOI:** 10.1097/MD.0000000000020970

**Published:** 2020-07-02

**Authors:** Yu Zhao, Kun Yin, Huiling Zhao, Zeli Peng

**Affiliations:** The First Affiliated Hospital of Dali University of Orthopedic trauma, Yunnan Province, China.

**Keywords:** femoral neck fracture, multiple cannulated screws, retrospective, sliding hip screws, study protocol

## Abstract

**Background:**

There has been a paucity of cohort trials directly comparing multiple cannulated screws (MCS) and sliding hip screws (SHS) in femoral neck fractures at any level. Thus, a well-conducted clinical trial with an adequate sample size is urgently needed. We undertake a retrospective study to compare outcomes in patients who undertake MCS or SHS fixation for femoral neck fractures.

**Methods:**

A retrospective review of femoral neck fractures performed with SHS or MCS between February 2016 and June 2018 was conducted with Institutional Review Board approval in the First Affiliated Hospital of Dali University of Orthopedic Trauma. All cases were performed by a single surgeon. Of these, we included 180 patients (90 hips) that were performed surgery in treatment of femoral neck fractures. All patients received the same standardized postoperative multimodal pain protocol and the same postoperative rehabilitation program. The primary endpoint was Harris Hip Score. Secondary outcome measures include operation time, length of hospital stay, incision length, patient satisfaction, and postoperative complications. Multivariate linear and regression analyses was used to identify independent predictors of outcome. A *P*-value of <.05 was defined as statistical significance.

**Results:**

We hypothesize that both treatments provide comparable outcomes.

**Trial registration:**

This study protocol was registered in Research Registry (researchregistry5638).

## 1 Introduction

A femoral neck fracture is 1 of the most common and devastating injuries encountered by orthopedic surgeons. Over 150,000 femoral neck fractures occur every year in the USA, and this number will double by 2050.^[1–3]^ In the Garden classification, Garden I and II fractures describe undisplaced femoral neck fractures in older patients.^[4,5]^ The treatment options are conservative (bed rest with or without traction) and surgical (internal fixation).^[6]^ Surgical treatment was reported to be optimal. However, any surgery is also associated with some risks.^[7]^

For the undisplaced femoral neck fractures, rigid fixation with early mobilization of the patients is the method of choice. Previously, multiple cannulated screws (MCS) or sliding hip screws (SHS) have been commonly involved to treat the undisplaced femoral neck fractures.^[8]^ Due to better biomechanical stability, antistress, and antirotation ability, osteosynthesis with MCS fixation is currently the preferred method for treatment of nondisplaced femoral neck fractures.^[9]^ Osteosynthesis with MCS fixation is a less invasive technique, with less soft tissue stripping. However, early loosening of the screws may occur if the lateral cortex of proximal femur is osteoporotic.^[8]^

SHS fixation is well established in the treatment of extracapsular fractures, and in many fractures, SHS is effective at allowing controlled collapse of the fracture with consequent mechanical stability leading to healing of the fracture.^[10]^ However, in some hip fractures, there is deficient bone to share load with the fixation device. Rather than controlled collapse along the line of the screw, the screw may cut out from the head of the femur leading to failure of the fixation and damage to the hip joint. Revision surgery, to either refix or replace the proximal femur, is complex, and the outcomes are poor in this frail group of patients.^[11–13]^

There has been a paucity of cohort trials directly comparing SHS and MCS in femoral neck fractures at any level. Thus, a well-conducted clinical trial with an adequate sample size is urgently needed. We undertake a retrospective study to compare outcomes in patients who undertake MCS or SHS fixation for femoral neck fractures. We hypothesize that both treatments provide comparable outcomes.

## 2 Materials and methods

### 2.1 Study design and population

A retrospective review of femoral neck fractures performed with SHS or MCS between February 2016 and June 2018 was conducted with Institutional Review Board approval in the First Affiliated Hospital of Dali University of Orthopedic Trauma (DL0010443). All cases were performed by a single surgeon. This study was also registered in the Research Registry (researchregistry5638). All patients with a fracture of the femoral neck sustained within the last 3 weeks, as diagnosed on a plain radiograph of the pelvis with both hips in anteroposterior view, falling in the age group of 16–60 years were taken as cases. The exclusion criteria were patients with polytrauma, life threatening injuries or with other injuries in the same limb; American Academy Of Anesthesiology class 3 or 4.

### 2.2 Interventions

Under regional or general anesthesia, closed hip reduction was ensured for patients in Group I under sterile conditions in the supine position followed by percutaneous fixation with 3 7.3 mm cannulated screws. The first screw was applied inferiorly in the femoral neck, the second screw near the posterior cortex and the third in the anterior side of the femoral neck; all screws were in parallel position. The proximal femur was exposed through the lateral approach in patients of Group II in the supine position. After fracture reduction under C-arm control without capsulotomy, fixation was achieved by SHS as in the original technique. One spongiosa screw was inserted as an anti-rotation screw.

### 2.3 Perioperative management

All patients received intravenous cefazolin sodium (1 g) and gentamicin sulfate (80 mg) before the operation and for 3 days after surgery. Low molecular weight heparin was administered to prevent deep vein thrombosis before the surgery and was continued for 21 days after surgery. All patients received the same standardized postoperative multimodal pain protocol, with 4 doses of 1 g of acetaminophen, 2 doses of celecoxib 200 mg, and morphine (first 48 hour) or tramadol (after 48 hour) for pain exacerbations. All patients underwent the same postoperative rehabilitation program. All patients were mobilized in the first day after the operation, without weight- bearing on the operated hip using crutches or walker. When follow-up radiographs showed sufficient healing and a pain-free hip was achieved clinically, patients were permitted controlled partial weight-bearing initially and full weight-bearing later, using crutches for 4 months.

### 2.4 Outcome evaluation

The primary endpoint was Harris Hip Score (HHS). HHS is valid and reliable and is often used as a reference/gold standard for assessing the construct validity of other patient-reported outcome measures for hip outcomes.^[14–17]^ HHS is a composite measure, with score ranging from 0 to 100, heavily weighted by pain and function; a higher score is better. It includes four domains: pain (1 item; 44 points), physical function (7 items; 47 points), deformity (5 items; 5 points), and range of motion (5 items; 4 points).

Secondary outcome measures include operation time, length of hospital stay, incision length, patient satisfaction, and postoperative complications. Satisfaction levels are rated using a 100 mm horizontal visual analog scale, for which 0 mm represent completely dissatisfied and 100 mm represent completely satisfied. Operation time, length of hospital stay, and incision length were obtained from our hospital database, as well as electronic and paper records. The HHS and postoperative complications were obtained both before and after surgery at a minimum of 2 years postoperatively.

### 2.5 Statistical analysis and power analysis

Statistical analysis was performed using Statistical Package for Social Sciences version 20.0 (IBM Corporation, Armonk, NY). Parametric and non-parametric tests were used as appropriate to assess continuous variables for significant differences between groups. A Student *t*-test was used to compare linear variables between groups. Dichotomous variables were assessed using a Chi square test. Multivariate linear and regression analyses was used to identify independent predictors of outcome (post-operative HHS). A *P*-value of <.05 was defined as statistical significance. A post-hoc power calculation was performed for the HHS: with 90 patients in the SHS group and 90 in the MCS group and a defined minimal clinically important difference of 5 points with a standard deviation of 9 and an alpha 0.05 achieved a power of 0.81.

## 3 Results

The results will be shown in Tables 1 and 2.

**Table 1:**
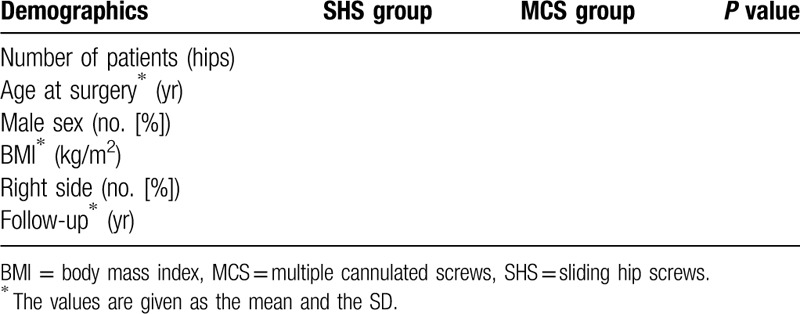
Patient baseline demographics.

**Table 2:**
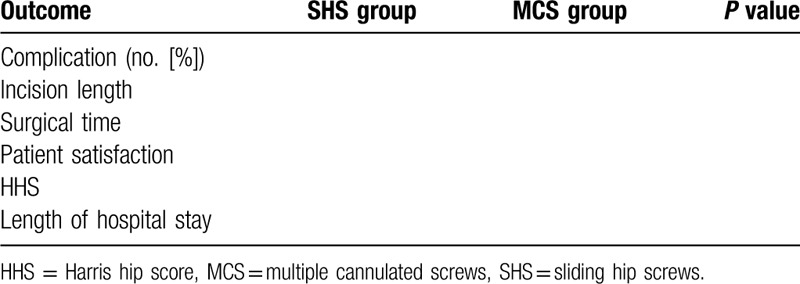
The outcomes in the 2 groups.

## 4 Discussion

Factors that affected the results after fixation of femoral neck fractures primarily depend on the condition of the patients, degree of fracture displacement, adequacy of internal fixations, and quality of surgical reduction. Different methods of internal fixation have an effect on the rates of union and osteonecrosis in femoral neck fractures. However, few studies have reported the clinical results of using the SHS and MCS. Therefore, there was a need for an evidence base or recommendations to help surgeons make clinical decisions.

The limitations of our study included those inherent in any retrospective cohort study, including the possibility of selection or observational bias. Most importantly, our sample size was small, with 180 patients. Second, the subjects may be exclusively Chinese. Therefore, the data from this clinical trial cannot be applied to other ethnic groups. A third limitation of this study is the mean follow-up period of only 2 years. Further follow-up is necessary and is underway.

## Author contributions

Yu Zhao and Kun Yin conceived, designed, and planed the study. Yu Zhao, Kun Yin, and Huiling Zhao are recruiting the study participants and performing the interventions. Zeli Peng supervised the study. Yu Zhao, Kun Yin, and Huiling Zhao will interpret and analyze the data. Yu Zhao drafted the manuscript. Yu Zhao and Zeli Peng critically revised the manuscript for important intellectual content. All authors have full access to the manuscript and take responsibility for the study design. All authors have approved the manuscript and agree with submission.
